# Trace Element Contents in Petrol-Contaminated Soil Following the Application of Compost and Mineral Materials

**DOI:** 10.3390/ma15155233

**Published:** 2022-07-28

**Authors:** Mirosław Wyszkowski, Natalia Kordala

**Affiliations:** Department of Agricultural and Environmental Chemistry, University of Warmia and Mazury in Olsztyn, Łódzki 4 Sq., 10-727 Olsztyn, Poland; natalia.kordala@uwm.edu.pl

**Keywords:** petrol contamination, compost, bentonite, calcium oxide, trace elements, soil

## Abstract

The global use of petroleum hydrocarbons as raw materials and an energy source in industry results in serious environmental, health, and ecological problems. Consequently, there is growing interest in the development of technologies for the rehabilitation of contaminated areas. This study was undertaken in order to determine the effect of different phytostabilising materials (compost, bentonite, and CaO) on the trace element content in soil contaminated with unleaded petroleum 95 (0, 2.5, 5, and 10 cm^3^ kg^−1^ of soil). The doses of petroleum applied to the soil were based on the previously conducted preliminary experiment. The highest petroleum dose (10 cm^3^ kg^−1^ of soil) significantly reduced the chromium, zinc, and cobalt contents in the soil. Petroleum increased the cadmium, lead, nickel, and copper contents in the soil. The materials used for phytostabilisation (compost, bentonite, calcium oxide) had a significant effect on the trace element content in the soil. The application of mineral materials (bentonite and calcium oxide) was more effective than the application of compost, compared to the control series (without soil amendments) as they reduced the contents of cadmium, chromium, nickel, and cobalt in the soil to the greatest extent. The reduction effect of bentonite and calcium oxide on the content of these trace elements in the soil was stronger than compost.

## 1. Introduction

Soil is a layer of the lithosphere, is biologically active, and serves numerous functions of environmental significance. It provides a place for the growth and development of plants and animals and transforms mineral and organic components [1]. The main functions of soil include: (1) the production of biomass, i.e., the basis of animal and human nutrition, and a source of energy and renewable resources [2,3]; (2) the participation in filtration, buffering and mineralisation processes, as well as the biochemical transformations of various substances [4]; and (3) maintaining a habitat for microorganisms and other soil organisms [5]. The soil absorbs and is an important source of greenhouse gases thereby influencing the composition of the atmosphere and thus the climate [6,7].

Among the most important characteristics of the soil are its sorption properties which allow it to retain (absorb or adsorb) various components including ions, particle suspensions, gases, vapours, and microorganisms [8]. Acting as a natural filter, the soil also stores and transforms harmful substances resulting from human activities [9]. Soil is a very slowly renewing resource and can take hundreds of years to form [10]. Both the industrial development and agricultural intensification put enormous pressure on the ability of a soil to maintain its function, thus resulting in the degradation of ecosystems and loss of productivity in the long term [11,12].

Crude oil is among the most important raw materials used in the energy and chemical industries. Petroleum-based products consist of aliphatic and aromatic hydrocarbons of different molecular lengths and non-hydrocarbon compounds, including nitrogen, sulphur, and trace elements [13,14]. Their spills during extraction, transport, and refining can cause severe environmental problems [15]. Global industrialisation results in widespread environmental pollution [16]. Crude oil and its derivatives are among the most important sources of anthropogenic contamination that penetrate the soil. The release of petrol into the environment may occur during transport, loading and unloading, following pipeline and tanker failures, uncontrolled emissions, war and political crises, sabotage, and natural disasters [17]. Soil contamination with petroleum products is a global problem due to their widespread use [18]. Once released into the soil, the above-mentioned substances spread to all ecosystems, thus posing a serious threat to human and animal health [19]. Contamination of soil with petroleum hydrocarbons changes the physical and chemical properties of the soil and has a phytotoxic effect on seed germination [20,21], crop growth, and yields [22,23]. This type of contamination has an adverse effect on the contents of oxygen, water, and nutrients (phosphorus and nitrogen) in the soil [18,19]. After entering the soil, these contaminants affect its permeability and porosity [24], as well as its biochemical and microbiological properties by reducing the counts of fungi and bacteria of the genera *Azotobacter* and *Pseudomonas* [25]. Additionally, these contaminants change the hydrophobicity of the soil and its water holding capacity by reducing it [26,27] and increasing the trace element content [28,29].

Moreover, they reduce the agricultural productivity of the soil [30,31] and cause the chemical contamination of groundwater, which limits its use and results in health and economic harm [19]. What is more, petroleum hydrocarbons have a harmful effect on soil enzyme functions and bacterial count [25,32]. They reduce the chlorophyll content in the leaves [33], interfere with the photosynthesis process [34], and can bind covalently to proteins, DNA, and RNA, thus disrupting the growth, metabolism, and enzymatic activity of plant cells [19]. For this reason, it is important to search for methods that will reduce the adverse impact of petrol on the soil properties and enable the efficient and cost-effective remediation of contaminated soils [35,36,37].

The removal of various types of contaminants from soils by thermal, chemical or electrolytic methods (hard technologies) may result in the loss of organic matter, the inhibition of growth and activity of functioning microorganisms or changes in the way living organisms are affected [38]. An interesting alternative to the physical and chemical methods that interfere with the environment are biological methods, including phytoremediation [39]. One of its variations is phytostabilisation, an environmentally friendly technique that is low cost, minimally invasive, and socially acceptable. The purpose of phytostabilisation is to reduce the migration of contaminants into the soil or across its surface with rainwater runoff through the introduction of materials supporting their immobilisation processes in the soil. The most commonly used soil additives, either alone or in combination, include organic composts, lime, bentonite, dolomite, fly ash, wood chips or bark, and phosphorous compounds [40]. Organic or mineral soil amendments change the speciation of trace elements and reduce their solubility and bioavailability by altering the pH value and the oxidoreduction potential of the soil [39,41]. These changes may result in the reduced availability of trace elements to less than phytotoxic levels. The main mechanism by which trace elements are deactivated and immobilised is through the precipitation of poorly soluble metal hydroxides and carbonates within the soil matrix [42,43].

Reclamation of soil with compost and mineral materials prevents the spread of contaminants in the soil and reduces the migration of trace elements through absorption and complexation, thus reducing their phytotoxic impact [44]. The introduction of these materials in the soil also improves its physical and chemical properties and fertility [45,46]. An important aspect in the development of contaminant phytostabilisation processes in the soil and the improvement of its quality is the search for inexpensive, available, and effective materials that can be used as soil amendments supporting the contaminant immobilisation processes. However, a study conducted as part of a vegetative pot experiment, and then under an environmental experiment may help optimise phytotechnology before its effective implementation.

In view of the above, the current study was conducted to determine the impact of petrol-contaminated soil on the trace element content in the soil following the application of various neutralising materials. It was assumed that the contamination of soil with a petroleum derivative would have an adverse effect by increasing the trace element content to potentially phytotoxic levels. The materials used for phytostabilisation included compost, bentonite, and calcium oxide.

## 2. Materials and Methods

### 2.1. Methodology of Vegetative Research

A vegetative pot experiment was conducted in a plant growth facility at the University of Warmia in Mazury, Olsztyn (northeast Poland) using the humus layer of Eutric Cambisol formed from sandy loam [47]. The basic properties of the soil were as follows: pH_KCl_—5.54; hydrolytic acidity (HAC)—23.2 mM kg^−1^; total exchangeable bases (TEB)—107.0 mM kg^−1^; cation exchange capacity (CEC)—130.2 mM kg^−1^; base saturation (BS)—82.2%; content of organic carbon (C_org_)—6.34 g kg^−1^, content of available phosphorus—29.32 mg kg^−1^, potassium—51.78 mg kg^−1^, and magnesium—62.48 mg kg^−1^. The experiment analysed the effect of increasing unleaded petroleum 95 doses (0, 2.5, 5 and 10 cm^3^ kg^−1^) on the content of trace elements in the soil. The doses of petroleum applied to the soil were based on the previously conducted preliminary experiment. Compost, bentonite, and calcium oxide were applied to the soil to reduce the potential adverse effect of petrol. Compost was used at 3% and bentonite at 2% in relation to the total soil weight, and calcium oxide (50% CaO) in a dose equivalent to one full hydrolytic acidity (1.08 g kg^−1^ soil). In addition, essential macro- and micronutrients were applied to the soil: nitrogen (N)—25 mg; phosphorus (P)—30 mg; potassium (K)—70 mg; magnesium (Mg)—50 mg; manganese (Mn)—5 mg; molybdenum (Mo)—5 mg; and boron (B)—0.33 mg kg^−1^ soil. Petrol, neutralising materials (compost, bentonite, and calcium oxide), and macro- and micronutrients were mixed thoroughly with 9 kg of soil before the experiment and placed in polyethylene pots. The trace element contents in the soil, compost, bentonite, and calcium oxide were provided in a previously published paper [48]. This was followed by the sowing of maize (*Zea mays* L.) of the Scandia variety. The plant density was eight plants per pot. During the plant-growing period, the soil-moisture content (i.e., 60% of capillary water capacity) was maintained at a constant level. Four replicates of the tests were conducted. At the maize tasseling stage, the plants were harvested and soil samples were collected for laboratory testing.

### 2.2. Methodology of the Laboratory and Statistical Analyses

The dried and sieved soil samples and neutralising materials were wet mineralised in a mixture of concentrated hydrochloric acid (HCl of analytical grade—1.18 g cm^−3^) and nitric acid (HNO_3_ cda—1.40 g cm^−3^). The mineralisation was carried out in a MARS 6 microwave digestion system (CEM Corporation, Matthews, NC, USA) according to the US-EPA3051 methodology [49]. The analysis of the trace element content (Cd, Pb, Cr, Ni, Zn, Cu, Mn, Fe, and Co) was carried out employing the atomic absorption spectroscopy (AAS) method [50] using a SpectrAA 240FS spectrophotometer (Varian Inc., Mulgrave, VIC, Australia). When analysing the trace elements in the soil, standard materials of the company Fluka (Cd 51994, Pb 16595, Cr 02733, Ni 42242, Zn 188227, Cu 38996, Mn 63534, Fe 16596, and Co 119785.0100) and the Certified Analytical Reference Material Soil S-1 (AGH University of Science and Technology, Kraków, Poland) were used.

The analysis of soil properties was carried out before the experiment using the following methods: pH of the soil using the potentiometric method in an aqueous 1 M dm^−3^ KCL solution [51]; hydrolytic acidity (HAC) and total exchangeable bases (TEB) using the Kappen’s method [52]; available phosphorus and potassium contents using the Egner–Riehm method [53]; and the available magnesium content using the Schachtschabel’s method [54]. Moreover, the cation exchange capacity (CEC) and the degree of soil-base saturation with alkaline cations (BS) were calculated using the following formulas: CEC = TEB + HAC; BS = TEB · CEC^−1^ · 100 [50].

The obtained study results were statistically processed using the Statistica package [55], including a two-way ANOVA analysis of variance, Pearson’s simple correlation coefficients, a principal component analysis (PCA) and the percentage of observed variability.

## 3. Results

The experiment results showed that soil contamination with petrol, as well as the soil amendments used, i.e., compost, bentonite, and calcium oxide, had a significant effect on the trace element content in the soil (Table 1, Table 2 and Table 3). The cadmium, lead, nickel, and copper contents in the soil in the series without neutralising materials were positively correlated with the increasing petrol doses. Compared to the control variant (not contaminated with petrol), the contents of these elements in the soil at the highest soil contamination level (10 cm^3^ kg^−1^) increased almost 2-fold for cadmium (r = 0.989) and lead (r = 0.999), by 17% for nickel (r = 0.647), and by 12% for copper (r = 0.872). In the same series, the contamination of soil with petrol contributed to a reduction in the contents of chromium (by 33%), zinc (by 19%), and cobalt (by 19%) in the soil in relation to the control object. The effect of petrol on the copper, manganese, and iron contents in the soil was not inconclusive. Based on the obtained results, it was also noted that the lowest petrol dose (2.5 cm^3^ kg^−1^) had a stimulating effect on the content of chromium, zinc, manganese, and iron in the soil, but a further increase in soil contamination had a reduction effect on the content of these elements.

The effect of neutralising materials (compost, bentonite, calcium oxide) on the trace element content on the soil varied and was determined by the analysed component and the level of petrol contamination. All (or most of) the soil amendments used increased the average contents of lead, iron, and (to a small extent) manganese in the soil while significantly reducing the content of cadmium, chromium, nickel, and cobalt (Table 1, Table 2 and Table 3).

Following the application of compost in the soil, the contents of cadmium, lead, chromium, nickel, and cobalt in the soil contaminated with the highest petrol dose (10 cm^3^ kg^−1^) were negatively correlated with the increasing doses of this oil-derivative substance. The greatest reductions in accumulation were demonstrated for cadmium (30%), lead (46%), and chromium (46%), when compared to the analogous object in the series without neutralising materials. Under the same conditions, the application of compost increased the content of zinc, copper, manganese, and iron in the soil by 48%, 17%, 14%, and 13%, respectively. On the other hand, in the non-contaminated objects, the introduction of compost in the soil contributed to an increase in the cadmium, lead, nickel, and zinc contents in the soil.

Soil remediation with bentonite in the objects contaminated with the highest petroleum dose increased the content of copper (by 16%) when compared to the control variant (without neutralising materials) while reducing the contents of cadmium, lead, chromium, nickel, iron, and cobalt in the soil. The greatest changes were noted for chromium and cadmium, whose contents in the soil decreased by 76% and 52%, respectively. In the non-contaminated objects, the introduction of bentonite significantly reduced only the chromium content (by 45%) in the soil while significantly increasing the concentration of lead (by 116%) and copper (by 27%).

In the soil with the highest petrol contamination level, the application of calcium oxide decreased the contents of nickel (by 11%), cadmium (by 26%), cobalt (by 33%), and chromium (by 55%) in the soil, when compared to the parallel object in the series without neutralising materials. As for lead, zinc, manganese, and iron, the correlation was inverse. The application of calcium oxide in the same series contributed to the content of the above-mentioned elements in the soil, with the greatest changes in the content noted for zinc (an increase of 28%). In the non-contaminated objects, the trend of changes in the contents of the analysed trace elements in the soil under the influence of phytostabilising materials was similar.

**Figure 1:**
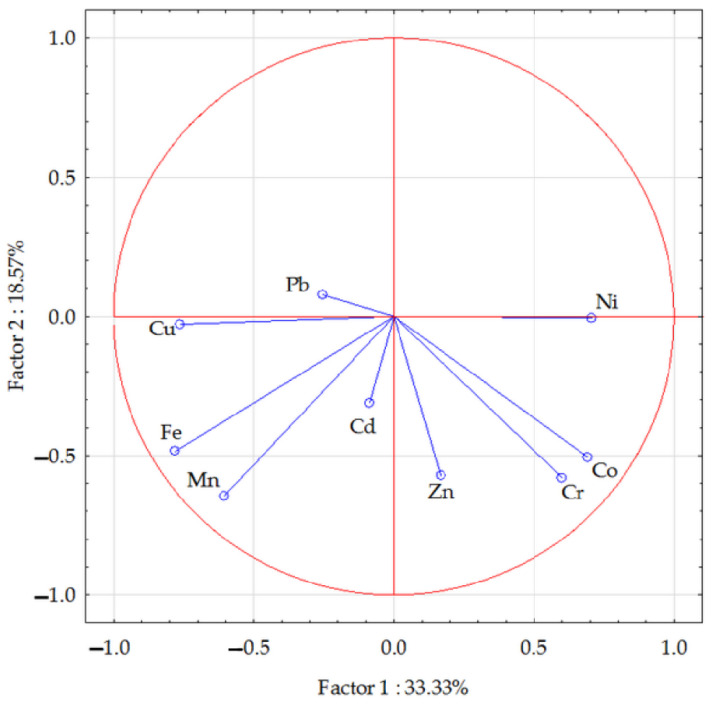
The content of trace elements in the soil illustrated with a PCA analysis. Key: vectors represent analysed variables (content of Cd, Pb, Cr, Ni, Zn, Cu, Mn, Fe, and Co).

**Figure 2:**
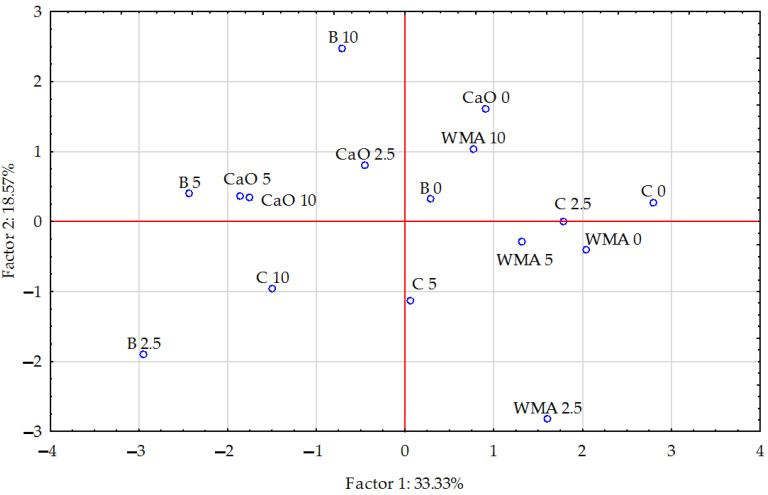
The effect of the amendments on the content of trace elements in the soil illustrated with a PCA analysis. Key: points show the samples with elements (WMA—without material amendments, C—compost, B—bentonite, CaO—calcium oxide; 0—0 cm^3^ (control), 2.5—2.5 cm^3^, 5—5 cm^3^, and 10—10 cm^3^ petrol per kg of soil.

The vector variables (Figure 1) confirmed the existence of significant correlations between particular trace elements in the soil. The PCA analysis showed changes in the contents of the analysed elements in the soil under the influence of petrol contamination and the phytostabilisation process. The total correlation of the data set for the first group of trace elements (chromium, nickel, copper, manganese, iron, and cobalt) amounted to 33.33%, while for the second group (cadmium, lead, and zinc) it was 18.57%. The strongest positive correlation was noted between cobalt and chromium as well as between iron and manganese + copper. The strongest negative correlation occurred between iron and nickel, and a weaker correlation between copper, nickel, and chromium. The vectors illustrating the cadmium and lead contents in the soil were shorter than the others, which may suggest the smallest contribution of these two components to the data set correlation. Based on the scattering of points in Figure 2, it can be concluded that the introduction of phytostabilising materials to the soil had a positive effect on the reduction in the contents of analysed trace elements in the soil.

**Figure 3:**
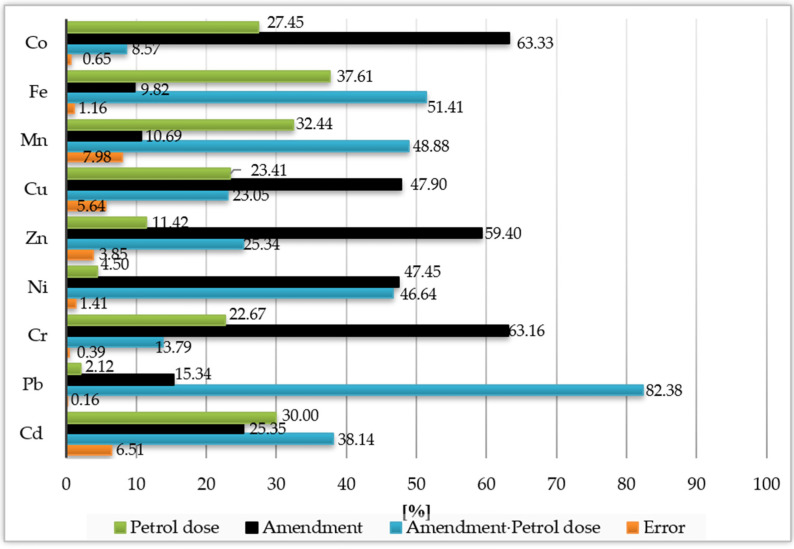
The percent contribution of variable factors according to the content of trace elements in the soil.

When determining the percentage of observed variability by the ANOVA method, using the η^2^ coefficient, it was found that the trace element contents in the soil were primarily determined by the type of neutralising materials used. This effect accounted for 63.33%, 63.16%, 59.40%, 47.90%, and 47.45% of the percentage of cobalt, chromium, zinc, copper, and nickel, respectively (Figure 3). For the remaining elements, lower values were demonstrated, which ranged from 9.82% (iron) to 25.35% (cadmium). The impact of soil contamination with petrol on the trace element contents in the soil was the lowest and mainly affected the contents of manganese (32.44%) and iron (37.61%).

## 4. Discussion

This study demonstrated that the contamination of soil with petrol at a dose of 10 cm^3^ kg^−1^ resulted in an increased content of cadmium, lead, and nickel in the soil compared to the control (non-contaminated) object. Other authors [56,57] have demonstrated that oil derivatives increased the cadmium, lead, copper, and manganese contents in the soil. This may explain the almost 2-fold increased cadmium and lead contents in the soil noted in this experiment. The concentrating effect of petroleum-derivative contamination on the lead, cadmium, and nickel contents in the soil was also reported by Onyeike et al. [58], Adeniyi and Afolabi [59], and Adesina and Adelasoye [60]. An increase in the mobility coefficients for trace elements (Cd, Cu, Pb, Ni, Cr, Zn, and Mn) and in their content in soils contaminated with crude oil compared to the control (non-contaminated) object was demonstrated by Iwegbue [61]. Qaiser et al. [62] assessed the impact of oil and gas exploration and extraction in Khyber Pakhtunkhwa (Pakistan) on soil contamination with trace elements. Those authors demonstrated a significant increase in the contents of lead, barium, and cadmium in the soil collected from areas influenced by the wastewater and discharges generated during the drilling process.

A possible reason for the increased trace element contents in the soil contaminated with petroleum compounds may be their acidifying effect. A reduction in the pH of the soil subjected to oil-derivative pressure was demonstrated by Akpan and Udoh [63] and Cheraghi et al. [64]. A reduction in the soil pH to slightly acidic and acidic increases the mobility of trace elements and their availability for plants and soil microorganisms [65]. In particular, high mobility is exhibited by cadmium, even in soils with a neutral and slightly alkaline pH [65] and in those accumulated with oil-derivative substances [66]. Cadmium is a more mobile element in soil and also more strongly taken by plants, compared to many other trace elements. This may explain its higher content noted in this experiment in the soil contaminated with incremental petrol doses.

In the series without mitigating materials, increasing doses of petrol reduced the contents of chromium, zinc, and cobalt while having no statistically significant impact on the contents of copper, manganese, and iron in the soil. The reduction in the content of chrome, zinc, and cobalt in the soil under the influence of petrol was correlated with an increase in the content of these trace elements in plants [48]. Zinc and, to a lesser extent, cobalt are more mobile and migrating in soils than other analysed trace elements. In addition, chrome and zinc are characterized by a greater degree of accumulation than manganese and iron. An analogous effect of soil contamination with crude oil derivatives on the contents of cadmium and lead as well as copper and zinc in the soil was obtained by Cheraghi et al. [64]. Oil-derivative substances entering the soil contribute to the occurrence of considerable amounts of oxidisable organic substances and to a change in the oxidoreduction potential of the soil. Under these conditions, copper (in its oxidisable phase) is hardly available as it forms stable bonds with the humus layer, complexed by humic and fulvic acids [67].

Carrying out reclamation involving the removal of contaminants using highly effective physical or chemical methods is, in many cases, unprofitable and labour intensive, and can degrade the biological activity of soil. What is more, the above-mentioned methods are devoid of the aspect of restoring the fertility of transformed land [68]. This results in a constant need to search for low-cost, effective, and technologically attractive techniques for the remediation of contaminated soils. Compared to other clean-up technologies, the application of soil amendments offers a number of advantages: (1) it reduces the mobility and bioavailability of trace elements; (2) it mitigates their impact on plants and groundwater; (3) it saves time and requires no change in the proper cropping sequence of individual plant species [69].

The mobility of trace elements in the soil environment is determined by their forms of occurrence and the mechanisms of binding with organic and inorganic components of the soil. The solubility of trace elements is strongly determined by the organic matter content in the soil, the cation and anion exchange capacity, the texture (soil content), the soil type, the redox potential, and the pH value of the soil [70]. For most chemical elements, their bioavailability decreases with soil alkalinisation. Under high pH conditions, the solubility of these elements decreases while their adsorption on soil colloids increases [71]. Therefore, the immobilisation of trace elements can be supported by raising the pH value of soil through liming [72,73], the application of organic matter (e.g., through composting) [74,75] or silty minerals which also serve the function of the contaminant absorbers [76,77]. The above-mentioned treatments are aimed at improving the sorption properties of the soil (an increase in the contents of humic substances or mineral fractions with ion-exchange properties) and immobilising metal ions through precipitation or formation of insoluble organometallic complexes with reduced phytoavailability [42,78].

Wyszkowski and Ziółkowska [79] noted the positive effect of compost, bentonite, and calcium oxide on the properties of petrol-contaminated soil. In general, all neutralising materials increased the pH, total exchangeable bases, and the degree of soil saturation with exchangeable alkaline cations while reducing the hydrolytic acidity. They also demonstrated that the effect of compost on the analysed soil properties, while positive in general, was weaker than bentonite or calcium oxide. The results of that study are in close correlation with those obtained in the current experiment.

In this study, soil remediation using compost and mineral materials contributed to a significant reduction in the average contents of cadmium, chromium, nickel, and cobalt in the soil. The obtained results can be confirmed by an experiment conducted by Radziemska et al. [80] where the authors demonstrated that the application of soil-applied substances in the form of mineral sorbents (dolomite, halloysite, chalcedony) and diatomaceous earth resulted in a significant reduction in the contents of chromium, nickel, zinc, and copper in soil contaminated with chromium. Shaheen et al. [81] also observed that the application of bentonite resulted in a considerable reduction in the labile fraction of nickel (58.7%) and zinc (83%) in the contaminated floodplain soil. Van Herwijnen et al. [82] proved that the application of green waste compost into soil derived from zinc smelting plant areas reduced the leaching of mobile forms of zinc and cadmium by 48%. On the other hand, Gusiatin and Kulikowska [83] report that the addition of sewage sludge compost reduced the bioavailability of cadmium, nickel, and zinc in the soil while having no effect on the lead and copper contents, which is partially consistent with the results obtained in this study. The differences noted in both experiments are likely to be due to a different composition of the compost used as well as the soil type and its granulometric composition.

In this study, bentonite and calcium oxide had a more favourable effect on the reduction in the trace element content in the soil contaminated with the highest dose of petrol (10 cm^3^ kg^−1^) than compost, thus resulting in the greatest reductions in the content of chromium (by 76% and 55%, respectively) and cobalt (by 24% and 33%, respectively). Comparable conclusions were presented by Malinowska and Jankowski [84], who indicated a stronger negative correlation between the application of lime (in the form of CaCO_3_) than sewage sludge and the cobalt content in the soil. The suitability of the liming process in the immobilisation of trace elements in the soil was also demonstrated by Cui et al. [85], Kosiorek and Wyszkowski [86], and Radziemska et al. [87]. The competition between Ca^2+^ and the trace element ions and their reduced mobility in soils with elevated pH reduces the contents of bioavailable forms of these elements and their rate of translocation to plant organs [88].

Bentonite is used in various industry sectors and consists mainly of silty materials. Due to its high water and sorption capacity [89], it can reduce the leaching of contaminants into certain environmental components following their absorption by the reactive surface [90]. Sun et al. [76] investigated the effect of the addition of bentonite to contaminated soils and proved that cadmium and lead contents in the soil with this material added were 11.1–42.5% and 20.3–49.3% lower than that in control soils. In addition, the application of bentonite caused an increase in the proportions of cadmium and lead fractions that were permanently bound in the soil.

The addition of bentonite also improves the soil’s ability to retain water and nutrients and accelerates emergence, the accumulation of the above-ground dry matter, and the rate of photosynthesis in plants [91]. It is used as a chemical and physical soil conditioner due to its high cation exchange capacity, which helps restore soil fertility and supports plant growth [92].

Even though the immobilisation of trace elements using various soil amendments does not remove them completely from the soil, it effectively reduces the ecological risks. However, in order to transform it into a cost-effective method of contaminated land reclamation, it requires further research, including under in situ conditions. The application of mineral and organic materials can be an effective instrument in reducing the impact of petroleum on the soil and plants.

## 5. Conclusions

The contamination of soil with petrol greatly modified the trace element content in the soil. In the series without phytostabilising materials, increasing doses of this oil-derivative substance (especially 10 cm^3^ kg^−1^ of soil) contributed to a decrease in the content of chromium, zinc, and cobalt in the soil. On the other hand, under the same conditions, the cadmium, lead, nickel, and copper contents in the soil were increased.

All of the tested materials neutralising the adverse impact of soil contamination with petrol have a significant effect on the trace element content in the soil. A limiting effect of most of these was noted for cadmium, chromium, nickel, and cobalt. The effect of compost, although generally positive, was weaker than that of bentonite or calcium oxide.

## Figures and Tables

**Table 1:** The content of cadmium, lead, and chromium in the soil (mg kg^−1^ d.m.).

Material	Petrol Dose (cm^3^ kg^−1^ d.m. of Soil)				Average	*r*
	0	2.5	5	10		
Cadmium (Cd)						
Without amendments	0.216 *^ab^*	0.270 *^bc^*	0.342 *^cd^*	0.414 *^d^*	0.311 *^A^*	0.989
Compost	0.270 *^bc^*	0.270 *^bc^*	0.288 *^bc^*	0.288 *^bc^*	0.279 *^AB^*	0.845
Bentonite	0.198 *^ab^*	0.306 *^bcd^*	0.306 *^bcd^*	0.198 *^ab^*	0.252 *^BC^*	−0.169
CaO	0.144 *^a^*	0.216 *^ab^*	0.216 *^ab^*	0.306 *^bcd^*	0.221 *^C^*	0.966
Average	0.207 *^I^*	0.266 *^II^*	0.288 *^II^*	0.302 *^II^*	0.266	0.884
LSD*_p_*_≤0.01_ for:	petrol dose—0.034, materials—0.034, interaction—0.069					
Lead (Pb)						
Without amendments	15.95 *^a^*	19.35 *^b^*	23.48 *^d^*	31.80 *^g^*	22.64 *^A^*	0.999
Compost	33.68 *^gh^*	32.40 *^g^*	22.55 *^cd^*	17.13 *^a^*	26.44 *^B^*	−0.959
Bentonite	34.40 *^hi^*	33.65 *^gh^*	32.08 *^g^*	21.15 *^bc^*	30.33 *^C^*	−0.941
CaO	15.95 *^a^*	25.93 *^e^*	28.88 *^f^*	35.90 *^i^*	26.66 *^B^*	0.958
Average	25.00 *^I^*	27.83 *^II^*	26.74 *^III^*	26.49 *^III^*	26.52	0.280
LSD*_p_*_≤0.01_ for:	petrol dose—0.56, materials—0.56, interaction—1.12					
Chromium (Cr)						
Without amendments	13.00 *^g^*	18.33 *^h^*	8.97 *^f^*	8.71 *^ef^*	12.25 *^A^*	−0.635
Compost	6.89 *^d^*	5.98 *^cd^*	5.98 *^cd^*	4.68 *^bc^*	5.88 *^B^*	−0.970
Bentonite	7.15 *^de^*	5.98 *^cd^*	3.25 *^ab^*	2.08 *^a^*	4.62 *^C^*	−0.955
CaO	6.76 *^d^*	6.37 *^d^*	4.16 *^b^*	3.90 *^b^*	5.30 *^B^*	−0.898
Average	8.45 *^I^*	9.17 *^II^*	5.59 *^III^*	4.84 *^IV^*	7.01	−0.864
LSD*_p_*_≤0.01_ for:	petrol dose—0.49, materials—0.49, interaction—0.97					

*r*—correlation coefficient. Values with different letters are significantly different at *p* ≤ 0.01: ^*I*–*IV*^ for the petrol dose, ^*A*–*C*^ for the material amendments, and ^*a*–*i*^ for the interaction between the petrol dose and the material amendments (ANOVA, Tukey’s HSD test).

**Table 2:** The content of nickel, zinc, and copper in the soil (mg kg^−^^1^ d.m.).

Material	Petrol Dose (cm^3^ kg^−1^ d.m. of Soil)				Average	*r*
	0	2.5	5	10		
Nickel (Ni)						
Without amendments	14.56 *^ab^*	17.03 *^cd^*	17.55 *^de^*	17.03 *^cd^*	16.54 *^A^*	0.647
Compost	21.45 *^g^*	20.41 *^fg^*	19.11 *^ef^*	14.04 *^ab^*	18.75 *^B^*	−0.979
Bentonite	15.08 *^ab^*	13.65 *^a^*	14.17 *^ab^*	15.86 *^bcd^*	14.69 *^C^*	0.519
CaO	15.73 *^bcd^*	15.60 *^bc^*	14.95 *^ab^*	15.21 *^abc^*	15.37 *^C^*	−0.691
Average	16.71 *^I^*	16.67 *^I^*	16.45 *^I^*	15.54 *^II^*	16.34	−0.951
LSD*_p_*_≤0.01_ for:	petrol dose—0.54, materials—0.54, interaction—1.09					
Zinc (Zn)						
Without amendments	29.44 *^bcd^*	33.57 *^defg^*	29.46 *^bcd^*	23.88 *^a^*	29.09 *^A^*	−0.766
Compost	34.90 *^efg^*	35.01 *^efg^*	36.55 *^g^*	35.32 *^fg^*	35.45 *^B^*	0.328
Bentonite	30.97 *^bcdef^*	30.84 *^bcdef^*	27.27 *^abc^*	26.92 *^ab^*	29.00 *^A^*	−0.880
CaO	28.64 *^bc^*	30.25 *^bcd^*	31.84 *^cdef^*	30.65 *^bcde^*	30.35 *^A^*	0.607
Average	30.99 *^I^*	32.42 *^I^*	31.28 *^I^*	29.19 *^II^*	30.97	−0.736
LSD*_p_*_≤0.01_ for:	petrol dose—1.37, materials—1.37, interaction—2.74					
Copper (Cu)						
Without amendments	2.58 *^a^*	2.80 *^ab^*	2.83 *^abc^*	2.90 *^abcde^*	2.78 *^A^*	0.872
Compost	2.60 *^a^*	2.80 *^ab^*	3.30 *^def^*	3.40 *^f^*	3.03 *^B^*	0.922
Bentonite	3.28 *^cdef^*	3.35 *^ef^*	3.30 *^def^*	3.35 *^ef^*	3.32 *^C^*	0.603
CaO	2.80 *^ab^*	2.85 *^abcd^*	3.20 *^bcdef^*	2.85 *^abcd^*	2.93 *^AB^*	0.185
Average	2.82 *^I^*	2.95 *^I,II^*	3.16 *^III^*	3.13 *^II,III^*	3.01	0.833
LSD*_p_*_≤0.01_ for:	petrol dose—0.14, materials—0.14, interaction—0.28					

*r*—correlation coefficient. Values with different letters are significantly different at *p* ≤ 0.01: ^I–III^ for the petrol dose, ^A–C^ for the material amendments and ^a–g^ for the interaction between the petrol dose and the material amendments (ANOVA, Tukey’s HSD test).

**Table 3:** The content of manganese, iron, and cobalt in the soil (mg kg^−1^ d.m.).

Material	Petrol Dose (cm^3^ kg^−1^ d.m. of Soil)				Average	*r*
	0	2.5	5	10		
Manganese (Mn)						
Without amendments	271.8 *^ab^*	299.9 *^b^*	272.6 *^ab^*	258.3 *^a^*	275.6 *^A^*	−0.573
Compost	271.7 *^ab^*	283.5 *^ab^*	287.0 *^ab^*	293.4 *^ab^*	283.9 *^AB^*	0.939
Bentonite	271.2 *^ab^*	342.8 *^c^*	291.3 *^ab^*	269.1 *^ab^*	293.6 *^B^*	−0.304
CaO	274.1 *^ab^*	282.2 *^ab^*	288.5 *^ab^*	295.1 *^ab^*	284.9 *^AB^*	0.975
Average	272.2 *^I^*	302.1 *^II^*	284.8 *^I^*	279.0 *^I^*	284.5	−0.061
LSD*_p_*_≤0.01_ for:	petrol dose—11.2, materials—11.2, interaction—22.4					
Iron (Fe)						
Without amendments	8307 *^cde^*	9190 *^ghi^*	8472 *^def^*	8225 *^bcde^*	8549 *^A^*	−0.357
Compost	7179 *^a^*	7472 *^a^*	9058 *^fgh^*	9333 *^ghi^*	8260 *^B^*	0.911
Bentonite	8174 *^bcd^*	9704 *^i^*	9768 *^i^*	7784 *^abc^*	8857 *^C^*	−0.309
CaO	7683 *^ab^*	8829 *^efg^*	9597 *^hi^*	9478 *^hi^*	8897 *^C^*	0.814
Average	7836 *^I^*	8799 *^II^*	9224 *^III^*	8705 *^II^*	8641	0.530
LSD*_p_*_≤0.01_ for:	petrol dose—180, materials—180, interaction—360					
Cobalt (Co)						
Without amendments	3.12 *^i^*	3.09 *^i^*	2.91 *^hi^*	2.52 *^ef^*	2.91 *^A^*	−0.976
Compost	2.82 *^gh^*	2.70 *^fgh^*	2.64 *^efg^*	2.40 *^de^*	2.64 *^B^*	−0.994
Bentonite	3.09 *^i^*	2.40 *^de^*	2.10 *^bc^*	1.92 *^ab^*	2.38 *^C^*	−0.896
CaO	2.19 *^cd^*	1.89 *^ab^*	1.86 *^ab^*	1.68 *^a^*	1.91 *^D^*	−0.928
Average	2.81 *^I^*	2.52 *^II^*	2.38 *^III^*	2.13 *^IV^*	2.46	−0.978
LSD*_p_*_≤0.01_ for:	petrol dose—0.076, materials—0.076, interaction—0.153					

*r*—correlation coefficient. Values with different letters are significantly different at *p* ≤ 0.01: ^*I*–*IV*^ for the petrol dose, ^*A*–*D*^ for the material amendments and ^*a*–*i*^ for the interaction between the petrol dose and the material amendments (ANOVA, Tukey’s HSD test).

## Data Availability

Data are available by contacting the authors.
